# Infants prenatally exposed to SARS-CoV-2 show the absence of fidgety movements and are at higher risk for neurological disorders: A comparative study

**DOI:** 10.1371/journal.pone.0267575

**Published:** 2022-05-04

**Authors:** Vania Aldrete-Cortez, Liliana Bobadilla, Silvia A. Tafoya, Aline Gonzalez-Carpinteiro, Fernanda Nava, Carlos Viñals, Elsa Alvarado, Rosa Mendizabal-Espinosa, María Eugenia Gómez-López, Luz A. Ramirez-Garcia, Alejandro Perez-Miguel

**Affiliations:** 1 Laboratory of Neuroscience and Cognitive Development, School of Psychology, Universidad Panamericana, Mexico City, México; 2 Hospital of Ginecology and Obstetrics Dr. Luis Castelazo Ayala, Instituto Mexicano del Seguro Social, Mexico City, México; 3 Psychiatry and Mental Health Department, Faculty of Medicine, Universidad Nacional Autónoma de México, Mexico City, México; 4 Cerebral Palsy Department, Instituto Nacional de Rehabilitación: Luis Guillermo Ibarra Ibarra, Mexico City, Mexico; 5 Social Research Institute, University College London, London, United Kingdom; 6 Nacer Temprano, Vivir en Grande, Civil Association, Estado de México, México; 7 Psychology Departament, Instituto Nacional de Perinatología: Isidro Espinosa de los Reyes, Mexico City, México; Hospital Sant Joan de Déu, SPAIN

## Abstract

Congenital viral infections are believed to damage the developing neonatal brain. However, whether neonates exposed to severe acute respiratory syndrome coronavirus 2 (SARS-CoV-2) show manifestations of such damage remains unclear. For neurodevelopment evaluation, general movement assessments have been shown to be effective in identifying early indicators of neurological dysfunction, including the absence of fidgety movements. This study compared the early motor repertoire by general movement assessment at three to five months of age in neonates who were or were not prenatally exposed to SARS-CoV-2 to determine whether infants prenatally exposed to SARS-CoV-2 are at risk of developing neurological disorders. Fifty-six infants, including 28 in the exposed group of mothers without vaccination who had no need for intensive care and likely had SARS-CoV-2 infection close to the time of pregnancy resolution and 28 infants in the nonexposed group, were videotaped to compare their detailed early motor repertoires, in which a motor optimality score-revised (MOS-R) was calculated using Prechtl’s method by using the chi-square or Mann–Whitney U tests. In the exposed group, 3 (11%) infants showed the absence of fidgety movements with a total MOS-R<14 points, and 3 (11%) other infants showed abnormal fidgety movements. Between groups, atypical body symmetry (p = 0.009) and MOS-R values were significantly lower (Z = -3.08, p = 0.002), with a large size effect (Cohen’s *d* = 0.97). The consequences of this new virus go beyond the health of the pregnant mother, and these consequences in some of the infants in the exposed group are likely not transitory because of the absence of fidgety movements between 3–5 months; thus, these babies are at increased risk of developing a serious neurological disorder.

## Introduction

Severe acute respiratory syndrome coronavirus 2 (SARS-CoV-2) infection poses a particular risk to pregnant women and neonates compared with older children and healthy young adults [1]. It has been suggested that all congenital viral infections can generate neurological disorders [2, 3]. Although such disorders, which can manifest as a reduction in fetal movements [4], have been observed in conjunction with irritability, poor suction, axial hypertonia and opisthotonos in infants exposed to SARS-CoV-2 [5]; there are also studies in which neonates exposed prenatally to SARS-CoV-2 do not develop neurological alterations [6]. Therefore, to date, no definitive answers have been found that explain the broad spectrum of the effects of SARS-CoV-2 on the central nervous system (CNS) and, as with SARS-CoV and MERS, it is not yet known what their impact on long-term brain development is [7, 8].

Complications that result from viral infections during pregnancy can be hard to detect; on the one hand, there is a range of expressed behaviors, from minor neurological dysfunction with difficulties in the organization of higher mental functions [9] to serious neurological disorders [10]. On the other hand, some obvious clinical manifestations may not appear until the infant is older [11]. To fully understand neurologic development, multiple neurodevelopmental assessments are necessary and ideally include electrophysiologic studies such as auditory evoked potentials [12]. However, most of the assessments, except for the general movement assessment, require a face-to-face evaluation, which makes it possible to evaluate the infant through a recording of spontaneous movement [10] this is advantageous during the COVID-19 pandemic. Therefore, the assessment of general movements worldwide has been shown to be effective for identifying early indicators of neurological difficulties, providing an opportunity for early intervention [10]. In this regard, the most powerful indicator of general movements is fidgety movements because their absence has been proven to have high predictive value regarding the development of adverse neurological outcomes, especially cerebral palsy [10, 13], with a specificity and sensitivity of 89% and 97%, respectively [14]. The high predictive power is probably due to these endogenous movements being conclusive in terms of the ontogeny of general movements and the involvement of not only the areas of the brain involved in motor control but also of neighboring areas involved in emotional and cognitive control and in the connectivity between multiple regions [15].

The latest version of the general movement assessment is named the detailed early motor repertoire assessment, which calculates the motor optimality score (MOS)-revised (MOS-R). This allows for semiquantification of the quality of general movements, namely, fidgety movements, and other movements and postures that are apparent at 3–5 months, thus enabling the specialist to document small changes in these movements [13]. The total performance of the detailed early motor repertoire is represented in the total MOS, and the literature has proposed, among others, two cutoff scores: <24 points for nonoptimal performance [3] and <14 points for a severe reduction in performance [13]. Various studies have shown that a lower total MOS is associated with minor neurological dysfunction at 7 to 11 years of age [16] and language development difficulties between 4 and 10 years of age [17], with lower scores on intelligence, attention, and visual-motor integration tasks between 5 and 7 years of age [18]. When the total MOS also involves the absence of fidgety movements, the neurodevelopmental outcomes are worse.

To the best of our knowledge, this is the first study to evaluate the effect of prenatal SARS-CoV-2 exposure on infant neurodevelopment using general movements as a strong early behavioral marker. Identifying children at risk of neurological disorders for timely intervention is urgently needed for all infants exposed to SARS-CoV-2 infection during pregnancy because the literature has proposed a risk for damage to their developing fetal CNS [19–25]. Therefore, the aim of this study was to assess whether infants prenatally exposed to SARS-CoV-2 are at risk of developing neurological disorder by evaluating the early motor repertoire at three to five months of age in neonates who were or were not prenatally exposed to SARS-CoV-2. This study stems from a larger study that looks at biopsychosocial factors that might have an effect on the neurodevelopment of infants exposed to SARS-CoV-2 infection during pregnancy.

## Method

### Study setting and design

This is an observational and comparative study. The sample was selected nonprobabilistically from the neonatal population of hospitals in Mexico City between May 2020 and March 2021 corresponding to the first and second Mexican waves of COVID-19 pandemic in one of the most affected cities worldwide. The study was approved by the local Research and Ethics Committee R-2020-785-151, CIP-PI-054-2020-1 and E2003. All parents in the study gave their written informed consent after having been informed in detail about the research project. The assessments were observational and nonintrusive to the infants, and the procedures were conducted in agreement with the Declaration of Helsinki.

### Participants

The study participants eligible for evaluation between 3 and 5 months of age post term were identified between hospital admission and delivery. The inclusion criteria for the exposed and nonexposed groups were singleton births, ≥36 weeks of gestational, no congenital, chromosomal, or structural abnormalities that became apparent after birth, no serious illness or complications during the pregnancy or delivery (such as perinatal asphyxia) and no need for intensive care for the mother or newborn. For both groups, the exclusion criteria were infant birthweight < the 3rd percentile at birth; parental history of psychiatric illnesses and/or psychoactive substance use as verified in the medical record; failure to complete the assessments; or parental withdrawal from the study. The infants in the exposed group were born to mothers without any COVID-19 vaccine and a RT–PCR-SARS-CoV2 positive (during the time between hospital admission and delivery) with one test. As part of the institutional strategy during the onset of the pandemic all the recruited mothers in this study were assessed for their risk of suffering from COVID-19 and the RT-PCR-SARS-CoV2 test has been taken from mothers who report in the past ten days one major symptom such as cough, fever, headache, dyspnea along with at least one minor symptoms at the time of admission such as myalgias, arthralgia, odynophagia, shivering, chest pain, rhinorrhea, polypnea, anosmia, dysgeusia or conjunctivitis [26]. The infants in the nonexposed group were born to mothers who, upon entering the hospital to give birth, were not suspected of having COVID-19 because they had no symptoms or history of contact with someone positive for SARS-CoV-2 or with COVID-19 symptoms. If the mother had a high risk of suffering COVID-19, RT–PCR-SARS-CoV2 tests were performed for both the mother and her newborn, and they were moved to an isolated ward. Then, if neither was at risk of loss of life, they were removed from the isolation ward and subsequently discharged.

#### Personal data of parents and newborns

The demographic data of the parents and newborns, as well as the maternal–neonatal medical history during pregnancy, were obtained from the hospital records. Data on the following demographic and clinical characteristics of newborns were collected: sex, type of birth, gestational age, weight, height and head circumference at birth and medical history. The data of the mothers included age, marital status, educational level, and medical history.

#### Early motor repertoire assessment

The early motor repertoire was evaluated according to Prechtl’s method by detailed early repertoire assessment that calculated a MOS-R value for 3- to 5-month-old infants [13]. The parents who consented to the study were asked to videotape their infants’ spontaneous movements for 3–5 minutes when their children reached between 3 and 5 months of post term age, which was calculated as conceptional age + weeks of life in compliance with the procedure described by Einspieler et al. (1997) [27]. Infants were fully awake and were not crying, fussing, or sucking on a pacifier; their arms and legs were bare, and they could move their limbs and trunks freely. The infants were in the supine position on a mattress, lightly and comfortably dressed and in a silent room without any intervention by the person who was recording. The video was sent by the parents and reviewed by two certified experienced pediatric physiotherapists with GM Trust certified as an advanced scorer. C.V. L y E.A. blinded to the infants’ clinical histories and grouping. In case of disagreement on an infant’s assessment, they reviewed that infant video to reach a consensus on the final score. If the video did not meet all these requirements, the parents were asked to make the recording again.

From these videos, detailed early motor repertoires were evaluated through the following indicators: 1) fidgety movements, which were labeled normal, abnormal, or absent; 2) the concurrent repertoire motor, namely, movements and postures that were apparent at 3–5 months; 3) the total MOS, based on the sum of all instrument values, which ranges from 5 (poor) to 28 points (optimal) [28]; and 4) performance level, which was defined in the literature by two cutoff scores for nonoptimal performance <24 points [3] and for severely reduced performance <14 points [13]. The MOS-R has been proven to be valid and reliable in several different populations, including infants exposed to prenatal ZIKA virus, infants born extremely preterm, and infants born at term [3, 17, 29]. The MOS-R was calculated according to five aspects of motor development: 1) fidgety movements, 2) movement patterns, 3) age adequacy of movement repertoire, 4) postural patterns, and 5) movement character; the aspect of fidgety movements had a maximum score of 12 points, while the other four aspects had maximum scores of 4 points [13]. Interobserver reliability was studied extensively and reported to be good to excellent, ranging from 0.87 to 0.91 [16], and intraobserver reliability was noted to be high, with intraclass correlation coefficients ranging from 0.80 to 0.98 [30, 31].

Finally, a phone call was made to explain the results obtained from the evaluation of the videos. The mothers of infants who scored low in the evaluation were advised to attend centers where they could monitor the neurodevelopment of their infants.

### Statistical analyses

The data were analyzed using SPSS software, version 23 [32]. The distribution of MOSs was evaluated by means of the Kolmogorov–Smirnov test, which showed a nonnormal distribution; therefore, nonparametric statistics were used for hypothesis testing. The characteristics of mothers and infants were described according to the level of measurement of each variable. Continuous variables are presented as the means and standard deviations, ordinal variables are presented as medians (Mdns) and interquartile ranges (IQRs), and categorical variables are presented as frequencies and percentages. Comparisons between the exposed and nonexposed groups were made using the chi-square test for categorical data, t tests or the Mann–Whitney U test when the assumption of a normal distribution was violated; this last test was also used to compare ordinal variables. The confidence intervals of the differences were calculated to complement the hypothesis tests. Additionally, for the total MOSs that did not assume a normal distribution, *Z* statistics were transformed to obtain the effect sizes as Cohen’s *d* values [33].

When significantly different characteristics were found between the groups, to establish comparability (of factors not related to the presence of COVID-19), in mothers and infants, the effects of particular variables were assessed on the global outcome (total MOS); for continuous and categorical variables, Spearman’s rho and the chi-square test, respectively, were used, and subsequently, this effect were controlled in multivariate analyses (generalized linear model). A generalized linear model with a robust estimator of maximum likelihood of main effects was employed to assess how predictors were associated with the total MOS (dependent variable). The total MOS was considered a dependent variable because it included the evaluation of fidgety movements, as well as other accompanying movements and patterns. For all analyses, p < 0.05 was considered significant in two-tailed hypothesis testing.

## Results

### Participants

Initially, 81 newborns met the inclusion criteria, and the parents of 16 infants did not agree to participate. Of those who agreed, six infants did not have completed assessments, one infant was eliminated because of lack of SARS-CoV-2 test results for the mother, and two infants were eliminated because their birthweight percentile was <3. At the end of the study, 56 infants were included, with 28 in the exposed group and 28 in the nonexposed group. 100% percent contracted SARS- CoV-2 infection in the third trimester of pregnancy because they were having active infection at the time of delivery. Also, all of mothers were symptomatic and presented mild to moderate symptoms consistent with COVID-19 according to [34]. None of them needed intensive care nor mechanical ventilation or died. All women were discharged at home without complications.

**Fig 1:**
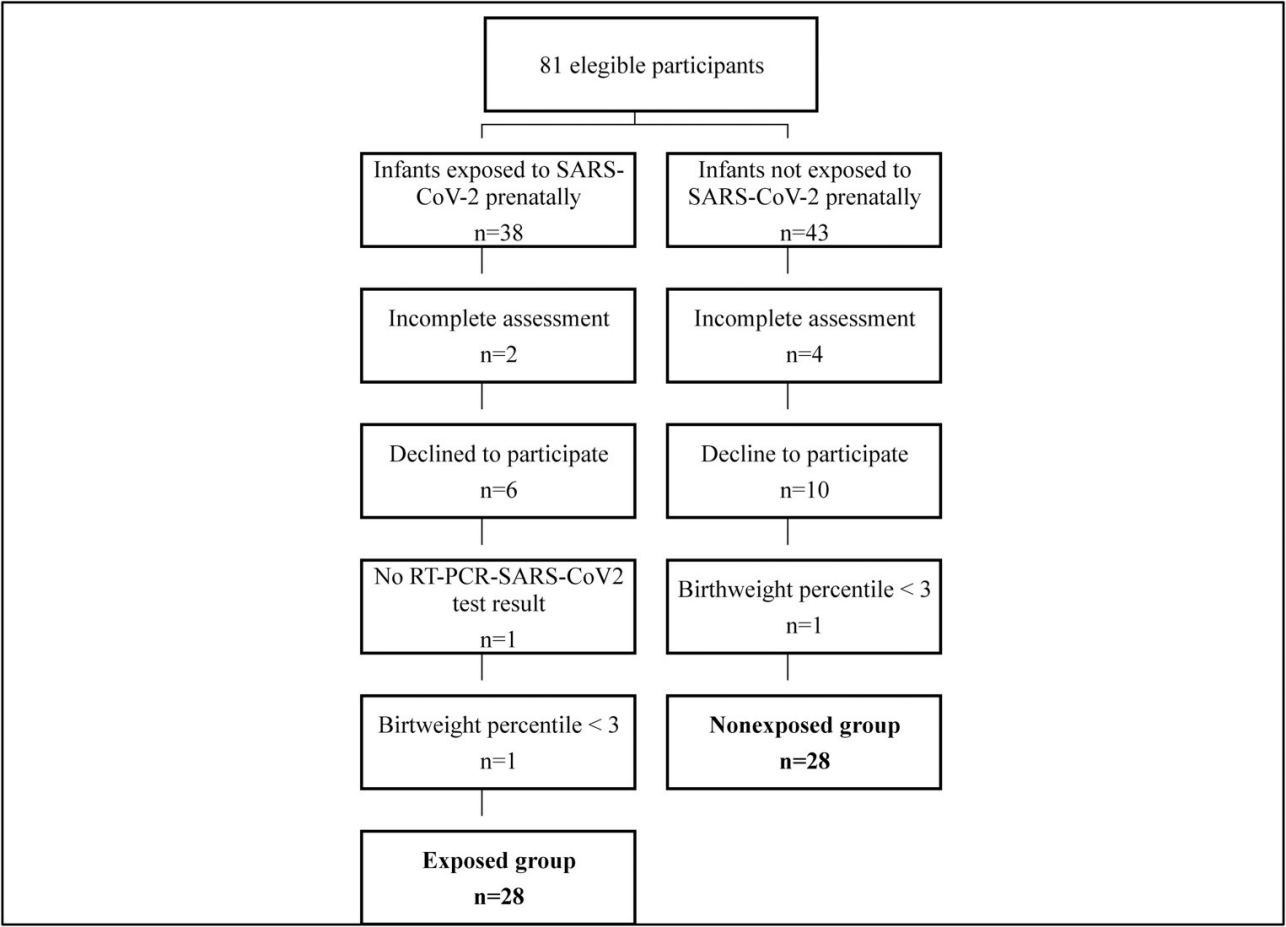
Study participants flow diagram.

The detailed exclusions by group are shown in Fig 1.

#### Comparison of mother’s and infant’s characteristics

No significant differences were found in maternal characteristics (see Table 1). In general, the mothers had an average age of 29.8±5.4 years, most had a partner (85%) and they reported having an educational level equal or higher to high school senior (82%).

**Table 1:** Comparison of the characteristics of mothers among the study groups.

	Exposed (n = 28)	Non-exposed (n = 28)	95% CI for the difference	*p*	Total
Age (years)^a^	29.8±5.6	29.9±5.3	[–2. 9, 3.0]	0.964	29.8±5.4
Marital status^b^*					
With partner	22 (82)	24 (89)			46 (85)
Without partner	5 (18)	3 (11)	[–0.11, 0.25]	0.442^§^	8 (15)
Education level^b^*					
≤ High school junior	3 (11)	7 (26)			10 (18)
≥ High school senior	25 (89)	20 (74)	[–0.02, 0.38]	0.139^§^	45 (82)
Preeclampsia^b^					
Yes	2 (7)	0 (0)			2 (4)
No	26 (93)	28 (100)	[–0.17, 0.02]	0.092^§^	54 (96)
Gestational diabetes^b^					
Yes	8 (29)	3 (11)			11 (20)
No	20 (71)	25 (89)	[–0.38, 0.02]	0.093	45 (80)
Hypothyroidism^b^					
Yes	6 (21)	2 (7)			8 (14)
No	22 (79)	26 (93)	[–0.32, 0.04]	0.119^§^	48 (86)

Note: CI = Confidence Interval.

^a^ Data reported as means ± standard deviations, analyzed by t test.

^b^ Data reported as frequencies (percentages), analyzed by chi-square test.

*Missing data.

^§^ Correction by likelihood ratio.

The sample of babies studied had a mean gestational age of 38.87±1.28 weeks, distributed similarly among sexes. The neonates in the two groups were similar in terms of most characteristics. However, differences were found in the type of delivery (χ^2^ = 10.50, p = 0.001) and length of hospital stay (*Z* = –3.04, p = 0.002) (see Table 2). Regarding the infant characteristics in the exposed group, the results of the newborn RT–PCR-SARS-CoV-2 test revealed that 24 (86%) were negative, 3 (11%) were positive, and 1 (4%) test result was not obtained. The effects of the different variables between the infant groups are analyzed later.

**Table 2:** Comparison of the characteristics of neonates among the study groups.

	Exposed (n = 28)	Non-exposed (n = 28)	95% CI for the difference	*p*	Total
Gestational age at birth (weeks)^a^	38.44±1.43	38.87±1.28	[–0.30, 1.16]	0.237	38.87±1.28
Sex^b^					
Female	15 (54)	11 (39)			26 (46)
Male	13 (46)	17 (61)	[–0.33, 0.19]	0.284	30 (54)
Type of delivery^b^					
Cesarean section	22 (79)	10 (36)			32 (57)
Vaginal birth	6 (21)	18 (64)	[–0.66, –0.20]	0.001	24 (43)
Hyperbilirrubinemia^b^					
Yes	2 (7)	1 (4)			3 (5)
No	26 (93)	27 (96)	[–0.10, 0.31]	0.549^§^	53 (95)
Apgar score at 5 minutes^c^	9 (9, 9)	9 (9, 9)	[0, 0]	0.643	9 (9, 9)
Stay in the hospital (days)^a^	3.39±2.73	1.60±2.10	[–2.81, 0.61]	0.002	2.54±2.18
Birthweight (g)^a^	3011±430	3100±502	[–163, 338]	0.485	3056±466
Length at birth (cm)^a^	48.75±2.10	49.30±2.18	[–0.60, 1.72]	0.377	49.02±2.14
Head circumference at birth (cm)^a^	34.12±1.32	34.20±1.54	[–0.74, 0.89]	0.920	34.16±1.41

Note: CI = Confidence Interval.

^a^ Data reported as means ± standard deviations, analyzed by t test.

^b^ Data reported as frequencies (percentages) and analyzed by chi-square test.

^c^ Data reported as medians (interquartile ranges), analyzed by Mann–Whitney U test.

^§^ Correction by likelihood ratio.

Because length of hospital stay and type of delivery were the only maternal and infant characteristics that were significantly different between groups, the effects of the group on the global outcome (total MOS) were assessed only for those factors were, first through simple tests: for length of hospital stay, Spearman’s rho was used, for type of delivery, the chi-square test was used, and subsequently, multivariate analyses (generalized linear model) were carried out. An association between total MOS and length of hospital stay was found (*rho* (56) = –0.31, p = 0.020). Moreover, there were no differences in total MOS between types of delivery (cesarean vs. vaginal) (*t* = –0.44, p = 0.663). Subsequently, to control for the possible effect of length of stay on total MOS, a generalized linear model of the main effects was also constructed. Here, total MOS was the dependent variable, and group (exposed or nonexposed) and length of stay in the hospital were independent variables. There were significant effects only for group on total MOS (omnibus test χ^2^ (2) = 12.91, p = 0.002, Table 3), that is, the exposed and nonexposed groups were comparable, and the possible differences in the early motor repertoire were not related to different perinatal factors after prenatal exposure to SARS-CoV-2.

**Table 3:** Group effect, adjusted for hospital stay, on the total MOS.

	B	β	*SE*	CI 95%	χ^2^ Wald	*p*
Intersection	19.70	–0.29	1.39	[16.96, 22.45]	198.33	0.0001
**Group (exposed)**	4.01	0.81	1.18	[1.48, 6.55]	9.64	0.**002**
**Length of hospital stay (days)**	–0.29	–0.04	0.23	[–0.67, 0.23]	0.90	0.343
(Scale)	19.33	0.78	3.65	[13.35, 28.00]		

*B* = Beta, not standardized; β = Beta, standardized; *SE*, Standard Error; CI, Confidence Interval.

Detailed early motor repertoire

#### Total motor optimality score (MOS)

A total MOS that considered all the subcategories evaluated was compared between the exposed and nonexposed groups, and the age during this assessment was similar between groups, with a mean age of 54.31 ± 2.14 weeks for the exposed group and 53.61 ± 1.92 weeks for the nonexposed group (*t* = 1.34, p = 0.183, see Table 4). There were significant differences between the groups in almost all subcategories of the detailed early repertoire assessment except movement patterns where there was a trend toward a difference in fidgety movements. The total MOS was significantly lower in the exposed group (Mdn = 21, IQR = 6–26) than in the control group (Mdn = 23, IQR = 15–28) (*Z* = –3.08, p = 0.002), with a large size effect (Cohen’s *d* = 0.97). Although 13 infants (46% range 24 to 28 points) in the nonexposed group and 6 infants (21% range 24 to 26 points) in the exposed group were observed to have optimal performance, the median total MOS in both groups represented a nonoptimal performance, as the scores were mildly reduced in the exposed group and control group. These results showed that the exposed group had lower performance than the nonexposed group in the assessment; however, there were some infants with optimal performance.

**Table 4:** The motor optimality score and motor repertoires between 3 to 5 months post term age in neonates’ groups.

	Exposed (n = 28)	Non-exposed (n = 28)	95% CI for the difference	*p*	Total
Fidgety movements^a^					
Normal	22 (78)	27 (96)	[–0.34, –0.01]		49 (88)
Abnormal	3 (11)	1 (4)	[–0.06, 0.20]		4 (7)
Absent	3 (11)	0 (0)	NC	0.057^§^	3 (5)
Movements patterns^a^					
Normal>abnormal	22 (79)	26 (93)	[–0.32, 0.03]		48 (86)
Normal = abnormal	2 (7)	0 (0)	NC		2 (4)
Abnormal>normal	4 (14)	2 (7)	[–0.09, 0.23]	0.151^§^	6 (11)
Age-adequacy^a^					
Present	1 (4)	8 (29)	[–0.43, –0.06]		9 (16)
Reduced	7 (25)	9 (32)	[–0.30, 0.16]		16 (29)
Absent	20 (71)	11 (39)	[0.07, 0.56]	0.011^§^	31 (55)
Postural patterns^a^					
Normal>abnormal	5 (18)	14 (50)	[–0.55, –0.08]		19 (34)
Normal = abnormal	11 (39)	9 (32)	[–0.17, 0.32]		20 (36)
Abnormal>normal	12 (43)	5 (18)	[0.01, 0.48]	0.025	17 (30)
Movement character^a^					
Smooth and fluent	4 (43)	12 (43)	[–0.51, –0.06]		16 (29)
Abnormal but no CS	24 (57)	16 (57)	[0.06, 0.51]		40 (71)
Cramped synchronized	0 (0)	0 (0)	NC	0.037^§^	0 (0)
Motor optimality score^b^	20.5 (6–26, 17.0–22.7)	23.0 (15–28, 21.0–26.0)	[1.00, 6.00]	0.002	21.0 (6–28, 20.0–25.0)

Note: CS = Cramped synchronized, NC = Not calculated, CI = Confidence Interval.

^a^ Data reported as frequencies (percentages), analyzed by Chi-square test.

^b^ Data reported by median (min–max, IQR), analyzed by Mann–Whitney U test (Z).

^§^ Correction by likelihood ratio.

It is noteworthy to describe the total MOS results of the three infants with positive RT–PCR-SARS-CoV-2 tests. These infants scored 20, 23 and 26 points in the total MOS, that is, the first two had a mildly reduced score, and the third had an optimal score. The total MOS results of the 3 infected infants in the exposed group were not different from the overall results of the exposed group (*Z* = –1.38, p = 0.192) or the nonexposed group (*Z* = –.531, p = 0.629). These results highlight that the lower total MOS in the exposed group is not due principally to the performance in the assessment of the infant with a positive RT–PCR-SARS-CoV-2 test.

#### Fidgety movements

One of the most important components in the detailed early repertoire assessment is the evaluation of fidgety movement because it is considered a strong early biomarker of neurological outcome. Therefore, to describe the differences in fidgety movements between groups, it is important to highlight the results of the subjects with alterations in fidgety movements. In the exposed group, 3 (11%) infants showed an absence of fidgety movements, and 3 (11%) other infants showed abnormal fidgety movements. These infants had the lowest total MOS (6 to 13 points) in the whole sample. In the nonexposed group, 1 (3.5%) infant showed abnormal fidgety movements and had a total MOS of 15 points. Therefore, these findings are the most worrisome because of their predictive value for developing neurological disorders, particularly in the 3 infants with an absence of fidgety movements and with lower total MOSs.

#### Movements and postures present at 3–5 months

In the detailed early motor repertoire assessment, it is important to evaluate not only the general movements such as fidgety movement but also the movement and postures that are apparent at 3–5 months. Almost all the subcategories of detailed early motor repertoire: age adequacy, postural patterns, and movement character were significantly different between groups. Each category is composed of many items that describe the profile of each subject, and many times, the differences are not equal between subjects. However, only atypical body symmetry was consistent and significantly different between the exposed and nonexposed groups [24 (86%) vs. 15 (54%), χ^2^ = 6.84, p = 0.009]. These results explain part of the profile that reflects the lower total MOS of the exposed group, and the recognition of atypical body symmetry in these infants would be useful in the clinical approach.

## Discussion

There is an urgent need to recognize the effect of SARS-CoV-2 on all aspects of health. Among those aspects is the effect of SARS-CoV-2 exposure during pregnancy on fetal brain development and long-term neurological outcomes. Therefore, follow-up programs should be implemented to lessen the consequences. The aim of this study was to assess whether infants prenatally exposed to SARS-CoV-2 are at risk of developing a neurological disorder. The early motor repertoires of the infants of mothers without any vaccination presumably infected with SARS-CoV-2 close to the time of pregnancy resolution were compared to those of infants without exposure to this virus. In this study, the most worrisome finding was that a significant number of infants in the exposed group had the absence of fidgety movements and that other infants in the exposed group had abnormal fidgety movements. Moreover, the exposed group had a significantly lower total MOS than the nonexposed group, with a large size effect. It is likely that infants prenatally exposed to SARS-CoV-2 will develop along a spectrum from possible serious neurological disorders to minor neurological disorders to less-than-perfect optimal performance. Therefore, these findings are not a definitive answer to the effects of SARS-CoV-2 on the CNS and their impact on neurodevelopment. Rather, these findings raise the fact that some of the infants prenatally exposed to SARS-CoV-2 are at high risk of developing neurological disorders that should be corroborated in future follow-up investigations.

We found that the exposed group had a significantly reduced total MOS (Mdn = 21, IQR = 6–26) compared with that of the nonexposed group (Mdn = 23, IQR = 15–28). While our results showed differences that seem subtle, the effect size measures highlighted the magnitude of the difference. This lower performance in total MOS is consistent with the little evidence to date that pointed to lower performance in the neurodevelopmental assessments of infants with maternal choline levels <7.5 who were prenatally infected with SARS-CoV-2 [35] and infants prenatally exposed to SARS-CoV-2, particularly infants who suffered longer infant–mother separation than controls [36]. The tendency toward a lower performance in different domains of neurodevelopment is consistent with our findings, even though those studies used parental questionnaries, in contrast to the assessment in this study. Additionally, there were differences in the mother´s characteristics among samples. For example, the studies noted above were conducted in one sample of mothers infected in the first trimester of pregnancy [35] and the other in subgroups infected in the first, second or third trimesters of pregnancy, with maternal complications [36]; in both studies, symptoms were rated moderate to severe. However, in this study, 100% of the mothers presumably were infected with SARS-CoV-2 in third trimester of pregnancy with symptoms rated as mild or moderated without maternal complications that require intensive care and without any COVID-19 vaccine.

This study, beyond pointing out the lower performance in the early motor repertoire, adds to the existing literature that some infants prenatally exposed to SARS-CoV-2 have a risk of developing minor neurological dysfunction or serious neurological disorders. In our study, we found that six infants in the exposed group showed fidgety movements with variation: 3 (11%) showed the absence of these movements, 3 (11%) showed abnormal movements, and only one infant in the nonexposed group showed abnormal fidgety movements. In particular, the absence of fidgety movements among the three infants was the most worrisome finding because the absence of fidgety movements is a strong predictive marker for later serious neurological impairments [10], and not only because of the absence of fidgety movements but also because these infants showed low quality and quantity of movements and patterns that accompany fidgety movements that resulted in a severely reduced performance per the early motor repertoire score per the total MOS. The other three infants in the exposed group and one in the nonexposed group with abnormal fidgety movements were also worrisome, but this marker is less effective in predicting the seriousness of neurodevelopmental outcomes, which might fluctuate from performance within the norm to minor neurological dysfunction or serious neurological impairments [10]. Our findings suggest alterations in the CNS, consistent with Zeng et al. (2021) who pointed out that some newborns prenatally exposed to SARS-CoV-2 have abnormalities on brain magnetic resonance imaging (MRI), such as abnormal white matter signals, delayed myelination, brain dysplasia and abnormal bilateral periventricular signals [37]. Also, with Buonsenso et al. (2022) [38] who pointed out in a follow-up study that only a small subgroup of children exposed to SARS-CoV-2 in utero presented abnormalities among these, 15% of children had retinal abnormalities, such as bilateral reduced attention at distance in six cases (30%) and reduced contrast sensitivity in three (15%). Furthermore, our findings are, to some extent, similar to those obtained for infants prenatally exposed to ZIKA, where only some infants had a severely reduced total MOS and where, in the context of ZIKA, this occurred only in those who developed microcephalia [3]. It is currently unclear which are the mechanisms that trigger the worst neurological outcomes in only some infants exposed prenatally to ZIKA [39], as it happens in our results that only some infants exposed prenatally to SARS-CoV-2 had a severely reduced total MOS.

In addition, three infants with a SARS-CoV-2 positive result at birth with mild to optimal performance per the total MOS were found in this study. Given this observation, one can hypothesize that the physiological response of the mother infected with SARS-CoV-2 during pregnancy might be more important than the infant being positive for SARS-CoV-2 at birth. Thus, without evidence of the specific mechanisms that might underlie the effects observed in this study, it is possible that it is principally the maternal physiological response to SARS-CoV-2 infection that has an effect during the development of the fetal CNS that is likely not transitory, as suggested by the following hypothesis. First, maternal SARS-CoV-2 infection may have pathogenic-specific effects by triggering the maternal immune response toward the infectious agent itself and activating a proinflammatory cytokine storm [19], which could increase the likelihood that the offspring will develop a psychiatric disorder, particularly a disorder with a neurodevelopmental component [20]. Second, the infection can damage the placenta through the uncontrolled release of proinflammatory cytokines, thus contributing to the dysregulation of fetal development processes and, via maternal hypoxia, producing pathological changes in placental structure [21] and decreasing fetal oxygenation levels [22]. Third, increased expression of angiotensin-converting enzyme 2 (ACE2) during pregnancy due to maternal hypoxia could generate a reduction in fetal–placenta blood flow and, therefore, a decreased supply of nutrients to the fetus, causing developmental alterations [25]. Fourth, obstetric complications, which can trigger a series of perinatal complications [19, 23], could interfere with normal neonatal brain maturation [24]; in some studies, this hypothesis should be ruled out because of the inclusion criteria of the sample, as in this study.

Additionally, two secondary findings are noteworthy to mention. First, in the exposed group, the concurrent motor repertoire had a high frequency of atypical body symmetry. The total MOS calculated in the detailed early motor repertoire assessment brings together all the elements evaluated, that is, not only fidgety movements but also the movements and patterns that accompany them, named concurrent motor movements [28], which generally has clinical value for intervention. Normally, fidgety movements and symmetric body posture enhance head control, which in turn enables the infant to better interact with his or her environment, which might create a better basis for both psychomotor development and positive engagement during interaction with the caregiver [40]. Thus, body asymmetry posture characteristics should also be considered during evaluation and timely intervention to enhance early neurodevelopment in infants prenatally exposed to SARS-CoV-2. Second, although the group prenatally exposed to SARS-CoV-2 had worse performance, we observed that the scores of both groups were below the cutoff point, which defines optimal performance [28]. Regarding neurodevelopment, the COVID-19 pandemic is characterized by low exposure to stimulation due to social restriction measures, which may not be suitable for all infants. However, even though many infants do not show optimal performance, lack of optimal performance is not synonymous with pathology or abnormalities [41]; rather, it seems that these young infants do not engage in their motor repertoire in the best manner to interact with the environment, and these infants will be highly dependent on environmental stimulation to compensate.

### Limitations and future directions

Much of the focus during the pandemic has concentrated on adult care, including pregnant women. It was deemed urgent to study the neurodevelopmental outcomes of infants exposed to SARS-CoV-2 during pregnancy, hence the study design. However, the cross-sectional nature of the study did not allow establishing causal relationships between the variables analyzed. While we found the Prechtl’s assessment of general movement highly effective in identifying the absence of fidgety movements as a predictor of serious neurological disorders, we recognize that perfect predictions of outcomes cannot exist. The brain is highly plastic during early infancy, and there are many factors that influence a child´s overall motor outcomes, including nutrition, environmental enrichment, parental involvement, early intervention, and other medical treatments [13]. However, this study is embedded into a larger project; a qualitative component includes interviews with women and explores the effect of increased maternal stress before, during and after birth and their experiences of caring for their baby in the first months of life; the results are reported elsewhere. The homogeneity of the sample, particularly regarding the certainty that the infants were prenatally exposed to SARS-CoV-2 close to the time of pregnancy resolution without any maternal vaccination or severe symptoms and with none of them needing intensive care, might be seen as a strength of the study. Additionally, there were no other important perinatal factors that could have influenced the results. Future research is needed to understand the relationship between the type of mother´s response to the virus and the mechanism that affects the infant and should attempt to determine the mechanisms by which some infants, even those positive for SARS-CoV-2, achieve good performance regarding neurodevelopmental assessments, as the three infants positive for SARS-CoV-2 in this study presented high scores, while other infants did not.

## Conclusions

Infants prenatally exposed to SARS-CoV-2 have a risk of developing along a spectrum from possible serious neurological disorders to minor neurological disorders to a less-than-perfect optimal performance, which should be confirmed in follow-up research. Timely implementation of follow-up programs for infants exposed to SARS-CoV-2 prenatally is urgently needed to identify children at risk. Early intervention programs are also necessary to lessen the magnitude of possible neurodevelopment impairments for this population. An instrument, such as the Prechtl’s assessment of general movements, might more accurately identify children at risk than parent questionnaires. Policy makers and healthcare practitioners should consider incorporating low-cost follow up evaluation like in the present report.

Even though the exposed group in this study had lower performance, we found less than optimal performance in both the exposed and nonexposed groups. Policy makers should also consider designing adequate information and support for healthcare professionals and parents, given the pandemic-related restrictions in the social environments of infants who are exposed.
